# High functioning, yet high suffering - the need to incorporate invisible struggles in adult ADHD diagnostic assessment/criteria

**DOI:** 10.3389/fpsyt.2026.1813029

**Published:** 2026-05-18

**Authors:** Ines Homem de Melo, Gustavo Franca

**Affiliations:** 1 Clínica do Quinto Andar, Porto, Portugal; 2 CRI Porto Ocidental, Instituto de Comportamentos Aditivos e Dependências (ICAD), Porto, Portugal; 3 Hospital de Magalhães Lemos, Centro Hospitalar Universitário de Santo António, Porto, Portugal

**Keywords:** ADHD (attention deficit and hyperactivity disorder), ADHD diagnosis in adults, masking, compensatory mechanisms, diagnostic assessment, high-functioning ADHD, lived experience, neurodevelopmental disorders

## Abstract

Current DSM-5 diagnostic criteria for adult Attention-Deficit/Hyperactivity Disorder (ADHD) rely predominantly on externally observable signs and measurable functional impairment. While prior literature has criticized the child-centric and male-centric biases embedded in this framework, less attention has been given to its sign-centric and impairment-centric orientation. In this perspective article, we argue that this emphasis creates a critical diagnostic blind spot: adults who maintain high academic, occupational, or social performance through compensatory strategies and masking, yet experience substantial internal suffering. For these individuals, impairment is not absent but concealed beneath sustained effort, perfectionistic overcompensation, and chronic self-monitoring. The resulting psychological burden, emotional exhaustion, anxiety, shame, cognitive fatigue, and diminished quality of life often remain invisible within behavior-based diagnostic systems. We contend that current criteria capture the end-products of ADHD (observable behaviors and overt dysfunction) while neglecting the upstream processes shaping lived experience. Phenomena such as mind-wandering, internal restlessness, time blindness, sensory over-responsivity, and effortful self-regulation are clinically meaningful yet insufficiently represented in formal criteria. Moreover, compensatory mechanisms and masking, despite their relevance to clinical presentation and diagnostic accuracy, are not systematically assessed. We propose a process-based expansion of adult ADHD assessment that incorporates subjective cognitive phenomena, explicit evaluation of masking and compensatory strategies, and recognition of subjective distress and effort-to-output imbalance as diagnostically relevant dimensions. Such a paradigm shift would enhance diagnostic sensitivity, reduce misdiagnosis and underrecognition - particularly among high-functioning adults and women - and promote more equitable access to appropriate care. Recognizing invisible struggles is essential to capturing the full heterogeneity and lived reality of adult ADHD.

## 1 Introduction

Current DSM-5 criteria for Attention-Deficit/Hyperactivity Disorder (ADHD) rely predominantly on observable behavioral signs and functional impairment. This framework reflects several historical biases (1). First, it is child-centric, having been developed primarily around pediatric presentations (2). Second, it is male-centric, as it prioritizes externally visible hyperactivity and disruptive behaviors that are more commonly expressed in boys, thereby marginalizing symptom profiles more typical of girls and women (3). Third, it is sign-centric, privileging externally observable behaviors while largely excluding the subjective, lived experience of individuals with ADHD and giving insufficient weight to internal cognitive symptoms, such as attentional dysregulation and executive dysfunction. Finally, it is impairment-centric, assuming that without clear functional impairment, there is nothing to diagnose or treat.

The first two limitations, child-centeredness and male-centeredness have been widely discussed in literature, particularly regarding their role in the underdiagnosis of adults, women, and individuals with predominantly inattentive presentations (4, 5). In contrast, the latter two limitations, the overreliance on observable signs and the exclusive focus on measurable impairment, have received far less attention. It is precisely these overlooked dimensions that we address in this commentary.

This sign- and impairment-focused approach fails to account for a substantial subgroup of adults with ADHD who maintain high academic, occupational, or social functioning at the expense of compensatory strategies and masking. Although these individuals may appear outwardly successful, they frequently endure a significant internal burden, including emotional exhaustion, chronic stress, shame, and perfectionistic overcompensation. Their impairment is invisible, not absent. Because diagnostic systems privilege what is externally visible and measurable, they often fall below diagnostic thresholds, leading to underrecognition and inadequate access to care.

The term “high-functioning ADHD” is used here pragmatically to describe individuals who maintain high levels of observable performance despite significant internal difficulties. However, this terminology has been increasingly criticized, particularly in the autism literature, for obscuring the lived burden of effort and for implying a simplistic dichotomy between “high” and “low” functioning (6). In line with these critiques, we use the term cautiously, emphasizing that apparent functioning does not necessarily reflect underlying impairment or subjective distress.

A useful metaphor for this hidden burden is the so-called “floating duck syndrome” From above, the duck appears to glide effortlessly and gracefully across the water, yet below the surface it must paddle vigorously to stay afloat. Likewise, many adults with ADHD maintain an outward appearance of competence or high functioning while exerting disproportionate, invisible effort to manage or hide their symptoms. The contrast between visible ease and concealed struggle encapsulates the diagnostic blind spots created by an excessive reliance on external signs and underscores the importance of integrating lived experience into clinical assessment.

We contend that integrating subjective distress and compensatory strategies into diagnostic assessment would significantly improve diagnostic accuracy, foster greater equity across clinical populations and capture more fully the heterogeneity of adult ADHD presentations. This paradigm change would ultimately prevent unnecessary negative outcomes and foster the pursuit of new therapeutic targets.

## 2 The current DSM-5 criteria for adult ADHD: too many signs, too few symptoms

In psychiatric assessment, the distinction between signs and symptoms is fundamental. Symptoms refer to the subjective experiences reported by the patient. Internal states such as mind-wandering, rumination, guilt, or cognitive fatigue are examples of symptoms that cannot be directly observed by others and thus rely on self-report for their identification. Signs, in contrast, are externally observable phenomena, such as restlessness, disorganization, impulsive actions, or apparent inattention, which can be noted by clinicians or informants independent of the patient’s introspective account.

Traditionally, the conceptualization of ADHD emerged from neurology (initially framed as “minimal brain dysfunction”) and pediatrics, rather than from phenomenologically oriented branches of psychiatry (7). This historical background might explain the enduring emphasis on observable signs over subjective symptoms within contemporary diagnostic frameworks.

Therefore, current criteria privilege behaviors that can be externally seen, counted, or reported by others, while giving limited attention to the internal, experiential dimensions of the disorder. In this respect, the prevailing model offers “many signs and few symptoms”: it gives primacy to measurable, outwardly observable behaviors while discounting key phenomenological experiences such as mind-wandering (8), daydreaming (9), hyperfocus, internal restlessness, proneness to boredom (10), internalized emotional dysregulation 11, sensory overload, and time blindness 12, among others. These internal experiences are not only clinically meaningful; they are strongly associated with impairment, predict long-term outcomes, and are often the primary reason for adults to present for evaluation in the first place. Mind-wandering has even been proposed as a potential endophenotype of ADHD (13).

A clear illustration of this mismatch appears in one of the DSM-5 inattentive criteria: “Often does not seem to listen when spoken to directly (e.g., mind seems elsewhere, even in the absence of any obvious distraction).” The phrase “does not seem to listen” reflects an external observer’s perspective, one that is far more applicable to young children who lack metacognitive insight and cannot reliably report their own internal states. Adults, by contrast, can often articulate their experience with far greater precision: they may acknowledge that they are not listening even when they appear attentive, or conversely, they may appear disengaged while internally following the conversation. In such cases, the assessment of the individual’s subjective mental experience provides more accurate and clinically relevant information than reliance on external behavior alone.

Another important phenomenon to consider to consider in the assessment of neurodevelopmental conditions is masking, which can be defined as the suppression or concealment of externally observable signs to conform to social expectations. Although masking has been extensively documented in the autism literature (14), it is increasingly recognized as a relevant and understudied phenomenon in ADHD (15).

In ADHD, masking may involve the inhibition of self-regulatory behaviors such as fidgeting, sometimes replaced by less visible movements (e.g., biting the gums), or the adoption of elaborate avoidance strategies aimed at concealing functional difficulties (such as parking several blocks away from an event in order to prevent others from noticing that the vehicle is cluttered with trash).

The performance of individuals with ADHD can be highly inconsistent. The same person may demonstrate exceptional abilities in a highly supportive or well-structured context while performing very poorly in an environment that is not adapted to their needs. At first glance, such excellence in specific domains can mislead observers. An example might be an individual who holds a PhD and excels in complex intellectual tasks but struggles to manage everyday responsibilities, such as household organization or family logistics. Given the stark discrepancy between high-level expertise in one domain and pronounced impairment in another, particularly when the latter is conventionally perceived as simpler, such individual may feel compelled to conceal these difficulties through masking. This might create a kind of “secret” or parallel life, that is, an internal reality of difficulties that many people would find hard to believe.

While masking may facilitate short-term social acceptance, it often comes at a substantial psychological cost. Sustained efforts to suppress natural behaviors can increase cognitive load, contribute to chronic stress, and have been associated with diminished self-esteem, heightened shame, and a sense of inauthenticity (16).

Importantly, masking complicates clinical recognition by reducing the visibility of symptoms relied upon by behavior-based diagnostic frameworks, potentially leading to underdiagnosis or misdiagnosis, particularly in adults and in populations already less likely to display overt hyperactivity.

## 3 Too much focus on functional impairment

Clinical assessment of adult ADHD has become heavily anchored in models of functional impairment, with diagnostic decisions largely contingent on observable disruptions in academic, occupational, or social performance. Contemporary frameworks emphasize objective outcomes, missed deadlines, job loss, academic underachievement, as the central indicators of clinical relevance. While functional impairment is undoubtedly important, an exclusive focus on overt performance deficits risks producing a narrow and incomplete understanding of adult ADHD. In production-centered societies, emphasis tends to fall on external productivity metrics, ignoring internal effort or psychological cost.

A significant subgroup of individuals with ADHD does not exhibit the typical impairment profile (17). Through a combination of compensatory mechanisms (often supported by environmental scaffolding, socioeconomic privilege, or above-average cognitive abilities), many adults sustain high levels of performance across multiple life domains. Their academic records may be impeccable; their careers stable; their productivity seemingly exemplary. Yet, this external functionality is often achieved at the expense of substantial internal suffering. These individuals frequently rely on perfectionism, hypervigilant self-monitoring, overpreparation, emotional suppression, and extensive time investment to maintain the appearance of competence. The resulting psychological cost might include chronic fatigue, anxiety, emotional exhaustion, pervasive self-blame, and diminished quality of life. This psychopathological profile remains largely invisible within current impairment-based diagnostic frameworks.

This framing highlights a critical blind spot: compensatory strategies. These strategies can be spontaneous, socially learned, or formally taught through therapy or coaching. They may range from adaptive (e.g., structured routines, external reminders, body double techniques) to maladaptive mechanisms (e.g., perfectionism, hypervigilance, exhaustive self-monitoring, and the investment of disproportionate time and effort, often described as reliance on “sheer force”). Crucially, their effectiveness fluctuates according to internal resources, environmental demands, fatigue levels, and emotional state, meaning that an individual’s apparent functionality may be fragile, situational (context-dependent), and effort-dependent.

In such cases, high functionality is not evidence of the absence of ADHD, but evidence of the relentless compensatory mechanisms required to counteract its core symptoms. Many adults describe that every task demands disproportionately more effort compared to the neurotypical peers. The true burden of the disorder is therefore not reflected in performance outcomes, but in the effort-to-output ratio, a variable seldom considered in formal diagnostic criteria.

This situation is inherently paradoxical. Greater impairment can become, in effect, advantageous, insofar as only individuals who fail visibly and repeatedly are granted access to diagnosis, validation, support, and treatment. Conversely, those who maintain outward success, even at the cost of significant internal suffering and distress, often remain undiagnosed, unsupported, and very often misunderstood.

Notably, this phenomenon often persists even after a formal diagnosis has been established. Many high-functioning individuals report that revealing their diagnosis is met with skepticism, frequently framed by questions such as how one can have ADHD while maintaining such good performance. As a result, individuals must not only process the psychological impact of the diagnosis itself (and the suffering that led them to it), but also feel obliged to justify or defend its legitimacy to others. This dynamic may further exacerbate impostor syndrome, which many report having experienced long before receiving the diagnosis (18). As the saying goes, “You can’t judge a book by its cover,” a reminder that outward competence often obscures the invisible effort and distress beneath the surface.

## 4 Judging the book by its cover

The DSM-5 criteria for ADHD primarily describe the end-product of the disorder: observable behaviors and functional impairment, rather than the upstream processes that shape how the condition is experienced and managed by the patient. Conceptually, one can view DSM-5 criteria as reflecting the equation:

### 4.1 End-product (as measured by DSM-5) = ADHD – (compensatory strategies + masking)

Despite their clinical significance, compensatory strategies and masking are not systematically assessed within current diagnostic frameworks (19). Their presence can lead to the exclusion of an entire subgroup of individuals from diagnosis: those who do not present overt, clinically significant impairment because they have developed potent compensatory or masking mechanisms.

This raises an important question: Should these individuals be diagnosed and treated? Clinical experience strongly suggests that they should. The absence of overt dysfunction does not imply an absence of clinically significant suffering; rather, it may indicate the presence of intense compensatory effort whose psychological cost warrants clinical attention.

In many cases, treatment serves not only to address core ADHD symptoms but also to alleviate the burden of compensatory strategies themselves, which may constitute the individual’s primary source of distress and the main reason for seeking mental health care. For example, excessive checking behaviors, rigid self-monitoring, or perfectionistic control, sometimes presenting as obsessive–compulsive–like symptoms may function as compensatory responses to attentional or executive difficulties rather than as primary psychopathology (20).

A diagnostic system that focuses narrowly on externalized functional impairment therefore fails to capture the full phenomenological spectrum of ADHD and risks neglecting the lived reality of many adults with the condition. This limitation is particularly salient for women, high-achieving individuals, and those benefiting from strong environmental supports, that is, individuals who perform exceptionally well in highly structured or well-adapted contexts while struggling silently in less supportive environments. Recognizing and addressing this discrepancy is essential for equitable diagnosis, appropriate treatment, and a more accurate understanding of adult ADHD.

### 4.2 Beyond the DSM-5: incorporating lived experience, subjective distress, masking and compensatory strategies in adult ADHD diagnostic criteria

Some potential modifications to current diagnostic frameworks could address this gap:

#### 4.2.1 Including new criteria with emphasis on subjective cognitive phenomena (more symptoms, fewer signs)

Such criteria would contribute to a more comprehensive diagnostic framework, by integrating the internal and phenomenological dimensions of ADHD rather than relying predominantly on externally observable behaviors. Phenomenological features such as spontaneous mind-wandering, maladaptive daydreaming, subjective internal restlessness, low tolerance to monotony or boredom, emotional dysregulation with predominantly internalized expression, sensory over-responsivity, and impaired temporal perception (“time blindness”) warrant consideration as potential diagnostic markers. These features appear less susceptible to masking and compensatory mechanisms and are increasingly recognized in empirical studies as core, and potentially more specific (even endophenotypic), manifestations of adult ADHD.

Although assessment of such phenomena requires metacognitive capacity for self-report, this is typically more accessible in adults than in children, allowing for more nuanced characterization of the disorder’s internal cognitive-affective landscape. Integrating these domains into diagnostic criteria could therefore enhance specificity and sensitivity, while mitigating the risk of underdiagnosis in high-functioning individuals whose external performance may obscure clinically significant impairment.

#### 4.2.2 Going beyond impairment

##### 4.2.2.1 Incorporating a criterion of subjective distress

In many disorders, the presence of significant subjective suffering, regardless of visible functional decline, is a legitimate diagnostic marker. ADHD could similarly integrate the internal experience of emotional and cognitive strain as part of the diagnostic evaluation. Recognizing such suffering as diagnostically relevant therefore promotes a more accurate, humane, and inclusive understanding of adult ADHD while preventing the misattribution of secondary suffering to other, allegedly primary, psychiatric disorders.

In our clinical experience, subjective suffering can at times be so pervasive and emotionally overwhelming that it may be misinterpreted as major depressive disorder. The persistent frustration, chronic self-criticism, feelings of inadequacy, and emotional exhaustion associated with unrecognized and untreated ADHD can generate a depressive-like affective state that mimics core symptoms of depression (21). However, in these cases, the negative emotions often stem not from a primary mood disorder but from the continuous struggle to cope with executive dysfunction, unmet expectations, and the cumulative burden of sustained compensatory effort.

A similar mechanism can be observed with anxiety, which may operate as a maladaptive form of self-medication. Some individuals report being able to overcome procrastination or initiate tasks only when driven by heightened anxiety and its associated adrenergic arousal. While this anxiety-driven activation (typically triggered by looming deadlines and the imminence of failure or consequences) may temporarily enhance performance, it comes at the cost of chronic physiological stress, emotional distress, and increased vulnerability to anxiety disorders.

Failure to recognize affective disorders (be it depression or anxiety) as emerging from ADHD may lead to misdiagnosis and suboptimal treatment, with clinical interventions targeting exclusively secondary emotional states rather than the underlying neurodevelopmental condition. In fact, studies indicate a significant risk of diagnostic overshadowing, whereby ADHD remains obscured by the affective condition. In such cases, clinicians may not pursue a differential diagnosis of ADHD, resulting in the negative outcome of leaving the disorder untreated.

We must also take into consideration the cognitive strain involved. Parallel to the criteria used in Obsessive–Compulsive Disorder (The obsessions and compulsions are time consuming or cause significant distress), ADHD criteria could incorporate the excessive time, effort, or cognitive load required for certain executive tasks. This disproportionate “effort-to-output” ratio has been described in models such as the Mental Effort Reward Imbalance Model (MERIM), which highlights how individuals with ADHD may experience a mismatch between the cognitive effort invested and the reward or outcome obtained (22). Incorporating this dimension into diagnostic criteria would provide a more accurate representation of the functional burden imposed by the disorder. In many cases, this reflects a specific form of compensation that may warrant consideration as a distinct diagnostic indicator.

##### 4.2.2.2 Including a criterion explicitly acknowledging compensatory strategies and masking

The DSM-5 Criterion C for Autism Spectrum Disorder states “Symptoms must be present in the early developmental period (but may not become fully manifest until social demands exceed limited capacities or may be masked by learned strategies in later life).”

An analogous criterion for ADHD would acknowledge its dynamic nature (recognizing that impairment is context-dependent and evolves across developmental stages) as well as the compounding influence of masking and compensatory strategies, as made explicit in the formula presented above. Such a criterion would prompt clinicians to assess for the presence of these strategies, thus preventing the underdiagnosis of high-functioning ADHD.

### 4.3 How could this criteria be operationalized?

#### 4.3.1 Improving clinicians’ ability to evaluate subjective cognitive phenomena

While DSM-5 criteria emphasize observable signs and impairment, it is important to acknowledge that clinical diagnostic practice typically includes a semi-structured interview, through which clinicians can access aspects of the patient’s subjective experience. Even brief assessments may yield valuable insights into internal cognitive and emotional processes. However, in the absence of structured guidance or formal criteria incorporating these dimensions, such information may remain underutilized or inconsistently integrated into diagnostic decision-making. Embedding these domains more explicitly within diagnostic frameworks could therefore enhance both reliability and clinical sensitivity.

Validated instruments already exist, notably mind−wandering scales such as the Mind Excessively Wandering Scale (MEWS) (23), on which individuals with ADHD score markedly higher than controls. There are also psychometric tools capturing maldaptive daydreaming, subjective internal restlessness, low tolerance to monotony or boredom, predominantly internalized emotional dysregulation, sensory over−responsivity, and impaired temporal perception, even though none were developed specifically for ADHD. Integrating these measures into routine assessment would enhance diagnostic accuracy and yield a more nuanced understanding of how ADHD symptoms are expressed subjectively.

#### 4.3.2 Systematically evaluating both subjective distress, compensatory mechanisms and masking – taking a process-based perspective

In recent years, growing empirical interest has been directed toward the identification and measurement of compensatory mechanisms in ADHD. This has led to the development of instruments such as the Compensatory ADHD Behavior Scale (CABS) (24), designed to quantify the extent to which individuals engage in compensatory behaviors to manage attentional and executive-function challenges. Preliminary validation studies have demonstrated promising psychometric properties, supporting its usefulness in research and clinical contexts.

Closely related to compensation is the phenomenon of masking, particularly salient in adults and frequently reported among women. Recognition of this phenomenon has prompted the creation of emerging assessment tools, such as the Adult ADHD Masking Measure (25), which remains in the experimental phase and has not yet undergone full peer-reviewed validation.

As this field evolves, additional instruments are expected to emerge, aiding the detection of less-visible forms of impairment and contributing to more nuanced diagnostic assessments that capture the hidden dimensions of adult ADHD. Notably, despite these advances, there is still no widely adopted ADHD-specific multi-item scale that isolates the subjective experience of effort—including sustained mental energy expenditure, cognitive fatigue, and the perceived effort required to maintain attention—as a distinct measurable dimension (26). Recent conceptual reviews on effort in ADHD underscore this as a significant methodological and diagnostic gap, highlighting the need for tools capable of capturing energetic cost as an independent clinical feature.

## 5 Conclusions

The end-product alone tells little about the process required to achieve it — much like the destination of a journey reveals nothing about the route taken, the detours required, or the effort needed to get there. Observable performance, therefore, offers limited insight into the underlying process required to sustain it. Routine clinical assessment should therefore incorporate a process-based evaluation, integrating both effort expenditure and subjective cost. Such inquiry allows differentiation between true functional competence and functioning maintained at high personal cost. Furthermore, comprehensive assessment must include systematic evaluation of subjective and phenomenological experiences that remain invisible to external observation.

Together, these extensions would enable clinicians to more fully capture the true complexity of adult ADHD. Such an approach would shift the diagnostic focus from a narrow emphasis on outward impairment to a more comprehensive appreciation of lived experience. In doing so, the diagnostic process would better recognize individuals who show significant suffering despite maintaining high external performance, thereby promoting greater diagnostic accuracy, improving access to appropriate interventions, and ensuring that the full spectrum of adult ADHD presentations receives equitable acknowledgment and care. Addressing the needs of these individuals constitutes not only sound clinical practice but also a matter of justice, as it acknowledges and alleviates suffering that would otherwise remain invisible, while reducing the well-documented personal and societal costs of adult ADHD.

Importantly, the study of compensatory mechanisms also offers a constructive and forward-looking perspective. Within the broad range of compensatory strategies, some are clearly maladaptive and closely linked to distress, whereas others are adaptive and may support resilience. Systematic investigation of adaptive compensatory strategies, as well as the environmental conditions that facilitate them, holds promise for identifying novel therapeutic targets. Such insights may inform interventions that not only reduce symptom burden but also strengthen protective factors, optimize functioning, and promote long-term well-being in adults with ADHD.

As a final note, the enrichment of adult ADHD criteria is essential, given the complexity of the disorder, its ever-changing fluctuations in response to contextual demands and compensatory strategies, and the numerous invisible struggles experienced by high-functioning adults. As Antoine de Saint-Exupéry reminds us, “What is essential is invisible to the eye.” As psychiatrists, we owe it to our patients to foster the expertise to perceive what lies beneath the surface in order to truly understand their challenges, strengths, and, on a more musical note, to see their true colors.

## Data Availability

The original contributions presented in the study are included in the article/supplementary material. Further inquiries can be directed to the corresponding author.
